# OfWRKY48 coordinates floral aroma biosynthesis by dual regulation of MEP pathway and carotenoid metabolism in *Osmanthus fragrans*

**DOI:** 10.1093/hr/uhag167

**Published:** 2026-04-24

**Authors:** Huan Ju, Meng-Yu Jiang, Ruo-Yu Ma, Si-Jia Liu, Lin-Lin Zhu, Lin-Lin Zhong, Qin-Lian Yang, Shi-Ling You, Wan Xi, Ya-Fei Tan, Ri-Ru Zheng

**Affiliations:** National Key Laboratory for Germplasm Innovation & Utilization of Horticultural Crops, Huazhong Agricultural University, Wuhan, China; College of Horticulture and Forestry Sciences, Huazhong Agricultural University, Wuhan, China; National Key Laboratory for Germplasm Innovation & Utilization of Horticultural Crops, Huazhong Agricultural University, Wuhan, China; College of Horticulture and Forestry Sciences, Huazhong Agricultural University, Wuhan, China; National Key Laboratory for Germplasm Innovation & Utilization of Horticultural Crops, Huazhong Agricultural University, Wuhan, China; College of Horticulture and Forestry Sciences, Huazhong Agricultural University, Wuhan, China; National Key Laboratory for Germplasm Innovation & Utilization of Horticultural Crops, Huazhong Agricultural University, Wuhan, China; College of Horticulture and Forestry Sciences, Huazhong Agricultural University, Wuhan, China; National Key Laboratory for Germplasm Innovation & Utilization of Horticultural Crops, Huazhong Agricultural University, Wuhan, China; College of Horticulture and Forestry Sciences, Huazhong Agricultural University, Wuhan, China; National Key Laboratory for Germplasm Innovation & Utilization of Horticultural Crops, Huazhong Agricultural University, Wuhan, China; College of Horticulture and Forestry Sciences, Huazhong Agricultural University, Wuhan, China; National Key Laboratory for Germplasm Innovation & Utilization of Horticultural Crops, Huazhong Agricultural University, Wuhan, China; College of Horticulture and Forestry Sciences, Huazhong Agricultural University, Wuhan, China; National Key Laboratory for Germplasm Innovation & Utilization of Horticultural Crops, Huazhong Agricultural University, Wuhan, China; College of Horticulture and Forestry Sciences, Huazhong Agricultural University, Wuhan, China; National Key Laboratory for Germplasm Innovation & Utilization of Horticultural Crops, Huazhong Agricultural University, Wuhan, China; College of Horticulture and Forestry Sciences, Huazhong Agricultural University, Wuhan, China; Panasia Aromatech Wuhan Company Limited, Wuhan, China; National Key Laboratory for Germplasm Innovation & Utilization of Horticultural Crops, Huazhong Agricultural University, Wuhan, China; College of Horticulture and Forestry Sciences, Huazhong Agricultural University, Wuhan, China

## Abstract

The flowers of *Osmanthus fragrans* are widely appreciated for their distinctive fragrance, predominantly derived from monoterpenes and apocarotenoid volatiles. However, the transcriptional mechanisms coordinating the production of these scent compounds remain poorly understood. In this study, integrated transcriptomic and metabolomic analyses of five cultivars with varying scent profiles identified OfWRKY48, a Group II-c WRKY TF. Functional studies demonstrated that transient overexpression and virus-induced gene silencing of OfWRKY48 significantly altered the accumulation of key scent terpenoids, including linalool, its oxides, and the apocarotenoids β-ionone, α-ionone, and dihydro-β-ionone. Mechanistic investigations revealed that OfWRKY48 activated key genes in the methylerythritol phosphate (MEP) pathway, including *OfDXS, OfCMK, OfTPS1,* and *OfTPS2*, thereby promoting linalool synthesis. Furthermore, OfWRKY48 upregulated upstream carotenogenic genes (*OfPSY, OfPDS2,* and *OfLCYb2*) and, in parallel, enhanced the expression of the carotenoid cleavage gene *OfCCD4*, collectively boosting the production of ionone derivatives. DNA-binding assays, including yeast one-hybrid (Y1H), luciferase reporter, and electrophoretic mobility shift assays, confirmed that OfWRKY48 bound to the promoters of *OfTPS1, OfCMK,* and *OfLCYb2*. These findings establish OfWRKY48 as a key regulator that orchestrates the biosynthesis of both monoterpene and apocarotenoid volatiles. Our work reveals a coordinated regulatory mechanism that synchronizes precursor supply in the MEP pathway with branching points in the carotenoid pathway, providing new insights into the complexity of floral fragrance regulation and suggesting a promising target for molecular breeding of aroma traits in horticultural crops.

## Introduction

*Osmanthus fragrans*, a renowned aromatic plant in China, produces flowers renowned for their unique fragrance. They are widely utilized in food, cosmetics, and healthcare products as ingredients [1, 2]. The floral volatile profile, comprising >125 compounds including terpenoids, benzenoids, and esters, has been extensively characterized [3–5]. Notably, terpenoids constitute ~80% of total volatiles in most cultivars, with β-ionone, dihydro-β-ionone, α-ionone, linalool, and linalool oxides, emerging as dominant aroma compounds due to their high odor activity values (OAVs >1500) and diverse floral and sweet notes [6, 7]. They also play important roles in attracting pollinators and mediating plant defense, thereby facilitating plant–environment interactions [8]. Beyond these ecological functions, linalool and its oxides possess a range of pharmacological properties, including anti-inflammatory, antiviral, antimicrobial, and antidepressant activities [9]. β-ionone, dihydro-β-ionone, α-ionone are important apocarotenoids in plants. β-ionone is a major volatile apocarotenoid with a distinctive violet-like aroma that has been extensively used in perfumes, cosmetics, soaps, and food flavorings due to its pleasant scent and stability. α-ionone is also an essential fragrance material with potential bioactive properties, including antioxidant and anti-inflammatory effects, relevant to functional foods and supplements [10]. Industrial applications primarily leverage their aromatic properties and bioactive potential, establishing these apocarotenoids as versatile compounds across the flavor, agrochemical, and health sectors.

*Osmanthus fragrans* cultivars are classified into three color-based groups: ‘Albus’ (light yellow-white), ‘Luteus’ (yellow), and ‘Aurantiacus’ (orange). The scent profiles of these groups differ significantly: ‘Albus’ cultivars are rich in sweet, fruity apocarotenoids like β-ionone, dihydro-β-ionone, α-ionone due to enhanced carotenoid cleavage [11–13] while ‘Aurantiacus’ cultivars emit apocarotenoids along with fresh, floral notes from abundant linalool and its oxides [5, 6]. This natural variation provides an ideal system for identifying regulators of scent divergence.

Linalool and its oxides, C_10_ monoterpene and monterpene oxides, are synthesized through the plastid-localized 2-C-methyl-D-erythritol 4-phosphate (MEP) pathway, a well-characterized route conserved across plants [14, 15]. This pathway generates the universal five-carbon precursors isopentenyl diphosphate (IPP) and dimethylallyl diphosphate (DMAPP) [16], which are condensed by prenyltransferases to form geranyl diphosphate (GPP), the direct C_10_ precursor for monoterpenes [17, 18]. GPP is subsequently converted into linalool by specific terpene synthases (TPSs), enzymes that have been identified in various plants including *Solanum lycopersicum* [19], *Freesia* × *hybrida* [17, 20], and *Rosa hybrida* [21, 22]. In *O. fragrans*, this biosynthetic step is primarily mediated by *OfTPS1/2/5/6/7* [7, 23, 24].

A parallel branch of the MEP pathway utilizes IPP and DMAPP to form the C20 geranylgeranyl pyrophosphate (GGPP), which serves as the indispensable substrate for carotenoid biosynthesis. In *O. fragrans* flowers, the dominant carotenoids are β-carotene and α-carotene. These compounds are cleaved by dioxygenases *OfCCD1* and *OfCCD4* to produce volatile apocarotenoids, such as β-ionone and α-ionone, which are key aroma contributors [11, 12, 25–28]. Dihydro-β-ionone, likely a modified derivative of β-ionone, is hypothesized to arise via enone reductase or ene reductase activity [29, 30]. This integrated network highlights the enzymatic interplay shaping *O. fragrans*’s signature aroma, bridging monoterpene and apocarotenoid metabolism through shared precursors and species-specific modifications.

While the functions of key terpenoid biosynthesis genes (*OfTPSs*, *OfLCYb*, *OfCCDs*) have been well characterized [5, 7, 25, 26, 31], these represent the execution level of the pathway. However, transcription factors operate at a regulatory tier, coordinating multiple structural genes simultaneously to provide a systems-level understanding of how complex traits like floral scent are integrated and controlled. Recent studies have shown that transcription factors, including members of the MYB, bHLH, ERF, WRKY, and MADS families, orchestrate the production of specialized terpenoids. These encompass volatile aromatics (such as monoterpenes and sesquiterpenes) and nonvolatile pigments like carotenoids, primarily through direct promoter binding or the formation of protein complexes [32–37]. For instance, in *Freesia hybrida*, the interaction between MYB21 and MYC2 transcription factors regulates terpene synthase gene expression and terpenoid emission [33]. Furthermore, WRKY transcription factors have been implicated in carotenoid biosynthesis pathways in *Camellia chekiangoleosa* [38]. In a related context, the transcription factor LcERF19 was shown to bind GCC box elements in the *LcTPS42* promoter, enhancing its activity and thereby increasing geranial and neral production [39]. Similarly, *VvWRKY70* acts as a repressor of norisoprenoid via direct downregulation of β-carotene hydroxylase 2 gene (*VvBCH2*) [36]. Besides the direct regulation, transcription factors can also form protein complex to coordinately control terpenoid formation. SlSCL3, a scarecrow-like subfamily transcription factor (TF) could positively regulate monoterpenes and sesquiterpenes, modulate the expressions of pathway genes by transcriptional activation without direct protein–DNA binding [40]. In *Zea mays*, the activation of ZmEREB92 on terpenoid phytoalexins was dependent on the interaction with ZmMYC2, which could directly bind to the promoters of pathway genes [41].

A prior genome-wide study has identified 154 WRKY transcription factors in *O. fragrans* and analyzed their potential association with aroma synthesis, providing a foundational inventory of this gene family [42]. Building on this genomic resource, our study aimed to discover and functionally characterize specific regulators from this family that are capable of coordinating the broader metabolic network underlying the complex aroma. In *O. fragrans*, several transcription factors have been implicated in floral scent regulation. For instance, OfMYB21, OfWRKY33 directly activated linalool synthase genes (*OfTPS2* and *OfTPS7*) [5, 43], while OfWRKY3, OfERF61, and OfMYB1R114 enhanced β-ionone synthesis by binding to the *OfCCD4* promoter [11, 12, 44]. Additionally, chromatin accessibility analyses have provided further insights into the regulatory mechanisms of key floral scent production [45]. Building on this foundation, our study aimed to discover regulators capable of coordinating the broader metabolic network underlying the complex aroma of *O. fragrans*.

Over the past decades, integration of multi-omics data such as transcriptomics and metabolomics has been proved to be informative tool to investigate important genes involved in specific secondary metabolism. For example, previous work demonstrated a regulatory network governing flavor formation in *Actinidia chinensis* [46]. Another study identified two transcription factors *AdNAC5* and *AdDof4* as key regulators mediating the expression of the key gene *AdFAD1* [47], thereby affecting the ester formation. Although transcriptome profiles have been utilized to identify the structural genes in terpenoid metabolism of *O. fragrans* flowers, the transcriptional regulation of key pathway genes remains largely unknown.

In this study, transcriptome sequencing of five *O. fragrans* cultivars with distinct linalool and ionone profiles, combined with differential gene expression and weighted gene coexpression network analysis (WGCNA), identified *OfWRKY48* as a novel TF strongly correlated with monoterpene and apocarotenoid accumulation. Functional validation revealed its dual regulatory role: OfWRKY48 fine-tunes the expression of linalool synthase genes (*OfTPS1/2*), the MEP pathway enzyme gene *OfDXS* (1-deoxy-D-xylulose-5-phosphate synthase) and *OfCMK* (4-diphosphocytidyl-2-C-methyl-D-erythritol kinase), enhancing precursor supply and linalool biosynthesis. Simultaneously, it activates *OfPSY* (phytoene synthase), *OfPDS2* (phytoene desaturase 2), *OfLCYb2* (lycopene beta-cyclase 2), and *OfCCD4* (carotenoid cleavage dioxygenase 4), elevating β-ionone, α-ionone, and dihydro-β-ionone production. Yeast one-hybrid (Y1H), luciferase (LUC) assays, and electrophoretic mobility shift assays (EMSAs) confirmed OfWRKY48’s direct binding to conserved W-box motifs (TTGACC/T) in the promoters of *OfTPS1*, *OfCMK*, and *OfLCYb2*. This work uncovers a WRKY-mediated regulatory node that synchronizes terpenoid and apocarotenoid biosynthesis, challenging the conventional single-pathway regulatory paradigm. Our findings provide actionable genetic targets for engineering floral fragrance complexity in aromatic crops.

## Results

### Terpenoids are dominant aroma active compounds in flowers of five *O. fragrans* cultivars

**Figure 1:**
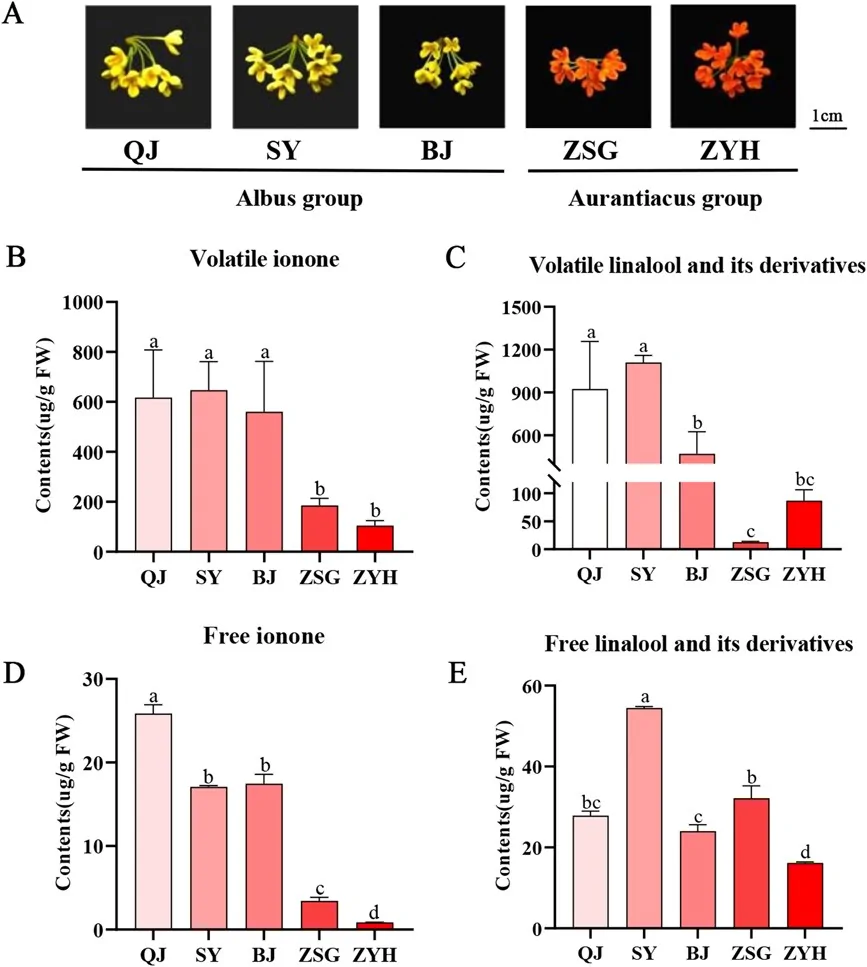
The aroma compound contents in *O. fragrans* flowers of different cultivars. (A) Full blossoming flowers of five *O. fragrans* cultivars. Cultivar 1: QJ (Qingjian), Cultivar 2: SY (Suiyin), Cultivar 3: BJ (Baijian), cultivar 4: ZSG (Zhushagui), cultivar 5: ZYH (Zhuangyuanhong). QJ, SY and BJ belong to the ‘Albus’ group, ZSG and ZYH belong to the ‘Aurantiacus’ group. Bars = 1 cm; (B) Volatile α-ionone, β-ionone, and dihydro-β-ionone contents of full-blossoming flowers in five cultivars detected by HS-SPME–GC–MS; (C) Volatile linalool and its oxides contents of full-blossoming flowers in five cultivars by HS-SPME–GC–MS; (D) Free α-ionone, β-ionone, and dihydro-β-ionone contents of full blossoming flowers in five cultivars by solvent extraction and GC–MS; (E) Free linalool and its oxides contents of full blossoming flowers in five cultivars by solvent extraction and GC–MS. All experiments included three biological replicates and were independently repeated three times. Data are expressed as mean ± SD. Statistically significant differences (*P* < 0.05, one-way ANOVA) among groups are indicated by different lowercase letters above the bars.

To investigate the aroma profile, we analyzed volatile and free aroma compounds in three ‘Albus’ and two ‘Aurantiacus’ cultivars of *O. fragrans* using head-space solid-phase microextraction (HS-SPME) and solvent extraction coupled with gas chromatography–mass spectrometry (GC–MS) (Fig. 1A). A direct visual comparison of the total ion chromatograms across these cultivars is presented in Supplementary Fig. S1.

We identified 66 volatile and 91 free aroma compounds (Supplementary Tables S1 and S2). The classification of these compounds revealed that terpenoids constituted the most dominant category, accounting for ~80% of the total volatile aroma compounds in the ‘Albus’ cultivars, and a substantially high proportion in the ‘Aurantiacus’ group as well. Correspondingly, the ‘Albus’ group accumulated significantly higher levels of both volatile and free terpenoids than the ‘Aurantiacus’ group (Supplementary Fig. S2). Notably, volatile compounds were far more abundant than their free counterparts, suggesting a tendency for high-volatile terpenoids to be released rather than stored (Fig. 1B–E).

Terpenoids dominated the volatile profile. OAV analysis further identified linalool and ionones as the key odorants among terpenoids due to their exceptionally high OAVs, which justified our focus on these compounds (Supplementary Tables S3 and S4). Among these, monoterpenes—particularly linalool and its oxides derived from the MEP pathway—were the primary aroma-active compounds in ‘Albus’ cultivars, with contents ranging from 469.7 to 1109.6 μg/g FW. In addition, the total content of volatile apocarotenoids—including α-ionone, β-ionone, and dihydro-β-ionone—was on average 4.2-fold higher in the ‘Albus’ group compared to the ‘Aurantiacus’ group (Fig. 1B–E).

To further validate the stability and reproducibility of the GC–MS data, we performed principal component analysis (PCA) based on the OAV values of major volatile compounds. The PCA score plot showed clear separation between the two cultivar groups along the first principal component (PC1), which explained 65.1% of the total variance. The three ‘Albus’ cultivars (QJ, SY, BJ) clustered closely, while the two ‘Aurantiacus’ cultivars (ZSG, ZYH) formed a distinct cluster, confirming consistent and statistically robust metabolic differences between groups (Supplementary Fig. S3A). Loading plot analysis identified linalool, β-ionone, dihydro-β-ionone, and linalool oxides as key contributors to this separation, consistent with our OAV and quantitative results (Supplementary Fig. S3B). This multivariate statistical validation supports the reliability of our GC–MS data and reinforces group-specific divergence in floral volatile profiles.

### DEG combined with WGCNA screen potential transcriptional factors regulating terpenoids

To elucidate the transcriptional underpinnings of these pronounced metabolic differences, we assessed the expression of key terpenoid biosynthetic genes. However, in the high-scent ‘Albus’ cultivars, their expression levels did not correspond to a consistent, across-the-board upregulation (Supplementary Fig. S4; Supplementary Table S5). This disconnect between dramatic volatile divergence and more subtle transcriptional variation at the structural gene level prompted us to search for upstream master regulators capable of orchestrating pathway flux.

To identify potential transcription factors regulating terpenoid biosynthesis in *O. fragrans*, transcriptomic profiling was conducted via Illumina NovaSeq sequencing of flower samples collected from five cultivars (‘Albus’ group: QJ, SY, BJ; ‘Aurantiacus’ group: ZSG, ZYH) at peak bloom period. Library preparation and high-throughput sequencing yielded a total of 180.19 Gb of clean data (15 libraries) across the Illumina HiSeq platranscription factororm. Sequence alignment against the reference genome of *O. fragrans* ‘Rixianggui’ [48] demonstrated high-quality data integration, with mapped reads ranging from 83.75% to 90.53% per library (Supplementary Table S6). All transcriptomes exhibited high-quality metrics (Q20 >98.86%, Q30 >93.66%, GC content 43%–45%), validating their suitability for further analyses.

**Figure 2:**
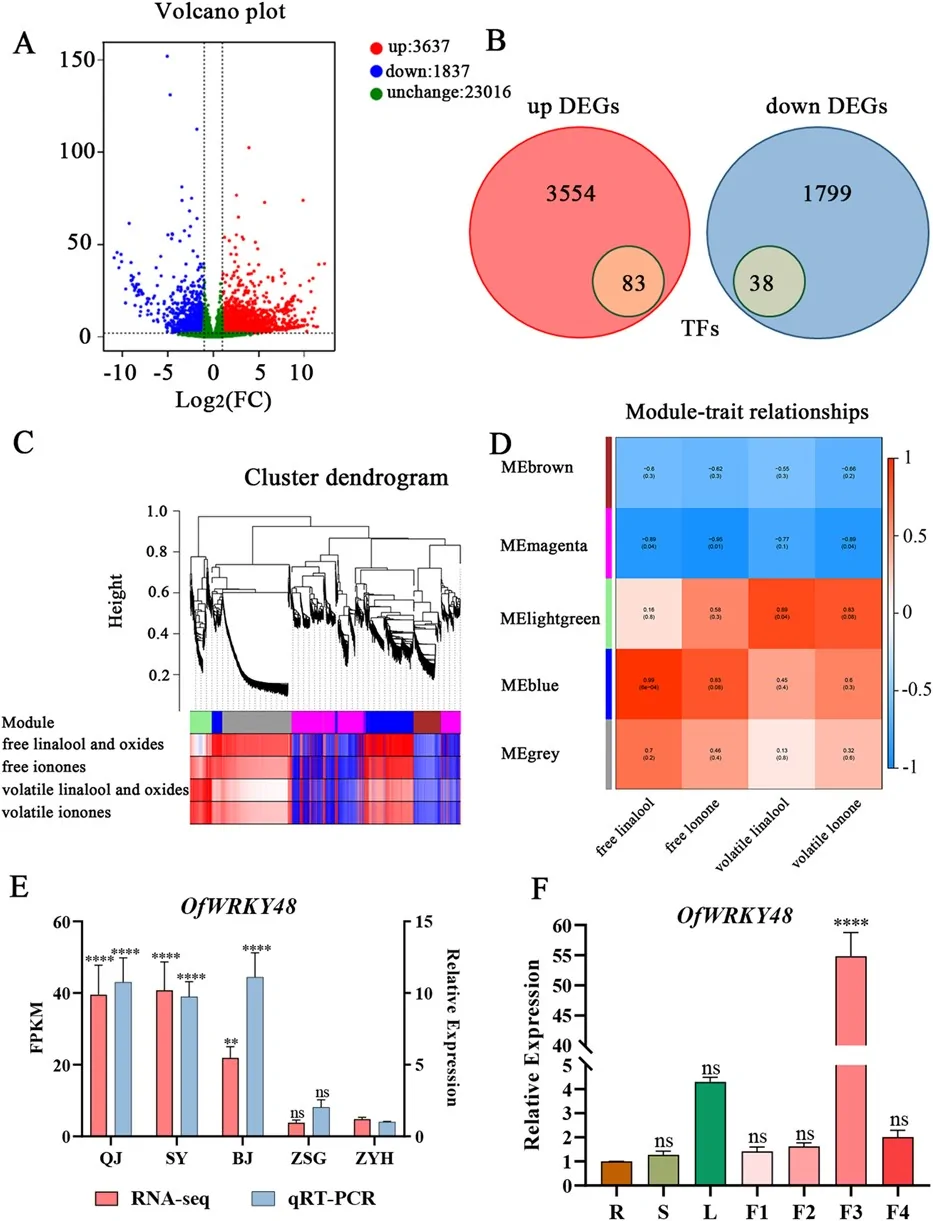
Screen of potential transcription factors regulating volatile linalool and its oxides according to transcriptome sequencings. (A) Volcano plot of DEGs identified between the ‘Albus’ and ‘Aurantiacus’ groups, including 3637 high-expressed and 1837 low-expressed genes in ‘Albus’ group. (B) Eighty-three high-expressed and 38 low-expressed transcription factors in ‘Albus’ group. (C) Dendrogram showing coexpression modules identified by WGCNA in different cultivars. The major tree branches constitute five modules labeled with volatile and free terpenoids. (D) Heatmap showing module–aroma compound correlations. Each row corresponds to a module indicated by different colors. Each column corresponds to aroma compounds. Positive correlations are shown in one tone, while negative correlations are shown in a contrasting tone. (E) Fragments Per Kilobase of exon model per Million mapped fragments (FPKM) derived from transcriptome sequencings and transcript levels determined by RT-qPCR of the potential transcription factor *OfWRKY48*. (F) Transcript levels of the potential transcription factor *OfWRKY48* in different tissues, respectively. R: root, S: stem; L: leaf; F1: bud; F2: initial bloom; F3: full bloom; F4: late bloom. All experiments were performed three times, each containing three biological replicates. The error lines represent the average ± SD. The asterisks represent significant differences compared with the control group (ZYH for panel E; R for panel F), evaluated by one-way ANOVA. ^*^P < 0.05; ^**^P < 0.01; ^***^P < 0.001；^****^P < 0.0001, ns: no significant difference.

Comparative transcriptome analysis between the ‘Albus’ and ‘Aurantiacus’ groups identified 5474 differentially expressed genes (DEGs) (|log₂FC| > 2, FDR < 0.01), with 3637 high-expressed and 1837 low-expressed in the ‘Albus’ group (Fig. 2A). Among these DEGs, 121 were transcription factors, with 83 high-expressed and 38 low-expressed in the ‘Albus’ group (Fig. 2B, Supplementary Table S7). Gene Ontology (GO) enrichment analysis categorized the 4325 DEGs into the three major categories: biological process, cellular component, and molecular function (Supplementary Fig. S5). KEGG pathway enrichment analysis mapped these genes to various metabolic pathways (Supplementary Fig. S6), with four terpenoid biosynthesis pathways being particularly prominent: sesquiterpenoid/triterpenoid biosynthesis (12 genes), diterpenoid biosynthesis (11 genes), monoterpenoid biosynthesis (10 genes), and terpenoid backbone biosynthesis (25 genes), underscoring the genetic foundation of terpenoid diversification in *O. fragrans* (Supplementary Table S8).

WGCNA of nonredundant genes identified five coexpression modules. Module–trait correlation analysis linked the light green module (*r* = 0.89, *P* = 0.04) and blue module (*r* = 0.83, *P* = 0.06) to volatile linalool and ionone accumulation, respectively (Fig. 2C and D; Supplementary Figs S7 and S8). One hundred twenty-eight transcription factors, primarily from the MYB, AP2/ERF, WRKY, NAC, bZIP, and bHLH families, were distributed in the light green and blue modules (Supplementary Table S9).

We integrated 121 DEGs with 128 transcription factors from WGCNA, resulting in an initial pool of 213 transcription factors. From these, 58 candidate transcription factors were identified using stringent thresholds (|log₂FC| > 2, FDR < 0.01) (Supplementary Fig. S9). This list was further refined to eight candidates that exhibited high expression (FPKM >10) in the ‘Albus’ group, a trend largely supported by quantitative real-time PCR (RT-qPCR) data across five cultivars, which showed generally higher expression in ‘Albus’ than in ‘Aurantiacus’ (Fig. 2E, Supplementary Fig. S10). Among these, six candidates demonstrated a consistent expression pattern between RT-qPCR and transcriptome data. Notably, *OfWRKY48* stood out, with RT-qPCR confirming its 3.2-fold higher expression in ‘Albus’ and showing strong concordance with the transcriptomic data (Fig. 2E). In addition, *OfWRKY48* was highly expressed in the full blossoming flowers compared to other tissues (Fig. 2F), suggesting its potential role as a central regulator of terpenoid biosynthesis in *O. fragrans*.

### OfWRKY48 is a nucleus-localized transcriptional factor

**Figure 3:**
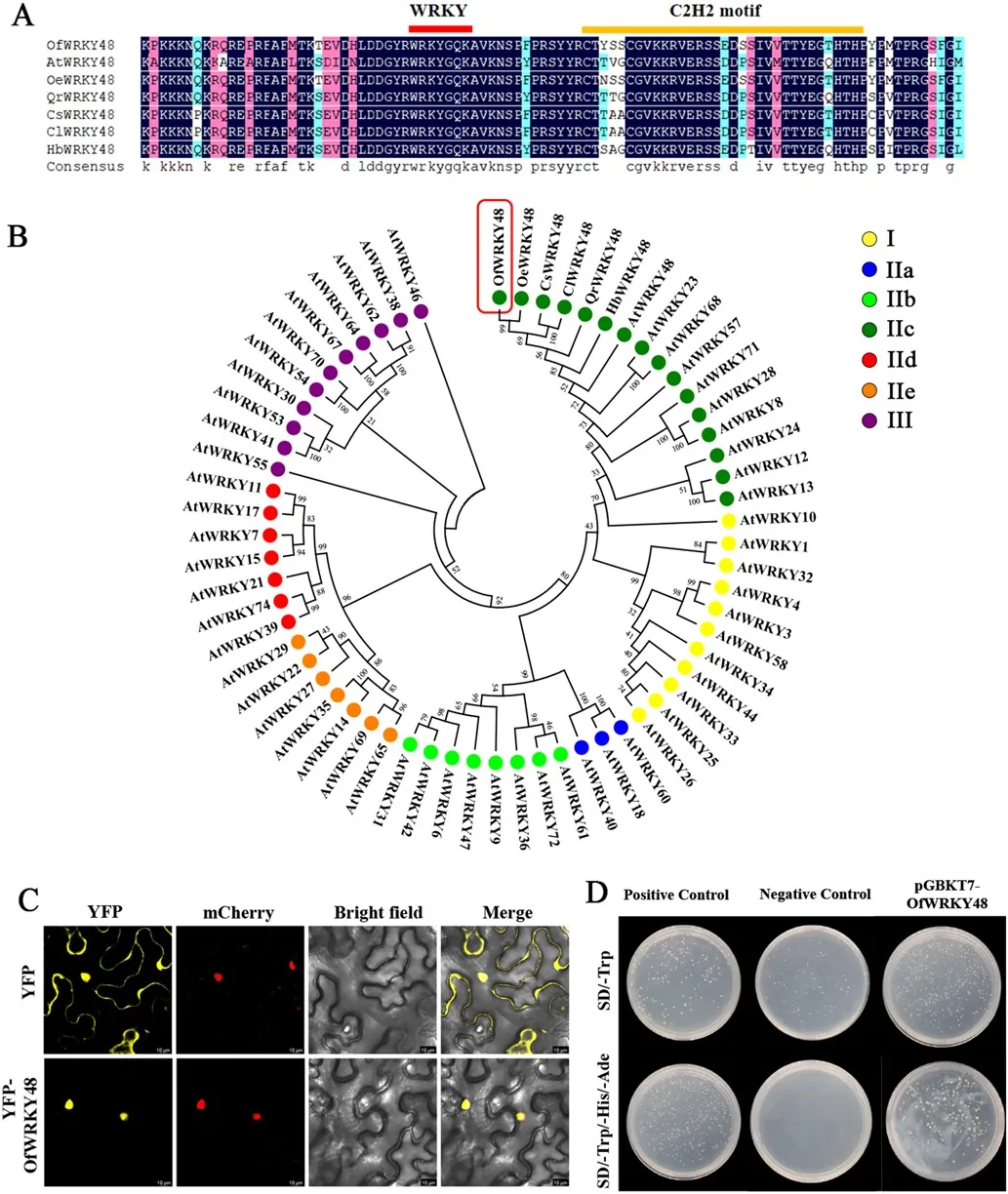
Characterization of OfWRKY48. (A) Multiple sequence alignment between OfWRKY48 and other plant WRKYs showing the conserved domains. (At: *Arabidopsis thaliana*; Oe: *Olea europaea*; Qr: *Quercus robur*; Cs: *Citrus sinensis*; Cl: *Citrus limon*; Hb: *Hevea brasiliensis*) The WRKY48 from other plant species were listed in Supplementary Data Table S7. (B) Phylogenetic analysis of plant WRKYs based on deduced amino acid sequences. The phylogenetic tree was constructed by using the maximum likelihood method through MEGAX software. The amino acid sequences of these WRKYs were listed in Supplementary Data Tables S10 and S12. (C) Subcellular localization of OfWRKY48 in *N. benthamiana* leaves. YFP signal (yellow) showed the localization of the fusion proteins, with the nucleus marked in red by the coexpressed nuclear marker. Bars = 10 μm. (D) Transcriptional activity of OfWRKY48 in yeast. Yeast strains harboring the indicated constructs were grown on synthetic dropout medium SD/-Trp/-His/-Ade. Positive and negative controls were pGBKT7-p53 + pGADT7-T and pGBKT7, respectively. The growth of the test group (pGBKT7-OfWRKY48) demonstrated the transcriptional activation activity of OfWRKY48.

The WRKY-like transcription factor contig *81.73-gene* exhibited high amino acid similarity with *OeWRKY48* from *Olea europaea* (CAA2957571.1), *CsWRKY48* from *Camellia sinensis* (AYA73392.1), and *ClWRKY48* from *Camellia lanceoleosa* (KAI8010938.1) (Fig. 3A, Supplementary Table S10), respectively, so it was designated as *OfWRKY48*. The full-length cDNA of *OfWRKY48* (GenBank: PX310283) contains a 951-bp open reading frame. The encoded protein comprises 316 amino acid residues and features the conserved WRKY domain (WRKYGQK) at its N-terminus and a C2H2 zinc-finger motif at the C-terminus (Fig. 3A, Supplementary Table S11). ProtParam predicted a coding sequence with a theoretical molecular weight of 35.78 kDa and an isoelectric point (pI) of 6.12. The grand average of hydropathicity (GRAVY = –0.928) indicated that OfWRKY48 is a hydrophilic protein. Secondary structure prediction via SPOMA revealed a composition of 24.37% α-helices, 2.22% extended β-strands, and 65.81% random coils (Supplementary Table S11). Tertiary structure modeling using SWISS-MODEL generated a 3D conformation consistent with these secondary structural elements, supporting the reliability of the predicted fold (Supplementary Fig. S11). The *OfWRKY48* gene was cloned from *O. fragrans* cv. ‘Rixianggui’. Comparative analysis of its coding sequence across the five studied cultivars revealed only a single, group-linked single-nucleotide polymorphism (SNP). This SNP is synonymous (encoding threonine in all cultivars), indicating that the OfWRKY48 protein sequence is functionally conserved (Supplementary Fig. S12).

To evaluate the evolutionary relationships, we generated a phylogenetic tree comparing *OfWRKY48* with *WRKY* genes from *Arabidopsis thaliana* and *WRKY48* orthologs from other species. *OfWRKY48* was grouped into the II-c subfamily and demonstrated the closest phylogenetic relationship with *WRKY48* orthologs from various cultivars (Fig. 3B, Supplementary Table S12). To determine the subcellular localization of OfWRKY48, the OfWRKY48-YFP construct was transiently expressed in *Nicotiana benthamiana* leaves. At 60 h postinfiltration, fluorescence imaging revealed that the OfWRKY48-YFP fusion protein was specifically targeted to the nucleus, as evidenced by its colocalization with the mCherry nuclear marker. In contrast, the control *CaMV35S:YFP* signal showed diffuse distribution throughout the entire cell (Fig. 3C). The transcriptional activity of OfWRKY48 was assessed *in vivo* by constructing a pGBKT7-OfWRKY48 fusion vector and introducing it into the yeast *Saccharomyces cerevisiae* strain AH109 Gold. The yeast strain with pGBKT7-OfWRKY48 and positive control vector could survive on SD (−Trp) medium and SD (−Trp/-His/−Ade), whereas the negative controls could not (Fig. 3D). Collectively, these results indicated that OfWRKY48 was a nuclear-localized protein and possessed transcriptional activity.

### OfWRKY48 upregulates terpenoid biosynthesis in *O. fragrans* through the MEP and carotenoid pathways

To elucidate the functional role of OfWRKY48 in terpenoid metabolism, we employed two complementary genetic approaches: transient overexpression and virus-induced gene silencing (VIGS) in *O. fragrans* flowers. The overexpression construct *PK7WG2D-OfWRKY48*, the silencing construct *pTRV2-OfWRKY48*, and corresponding empty vectors were introduced into *Agrobacterium tumefaciens* strain GV3101, which was then vacuum-infiltrated into floral tissues. Subsequent analysis of volatile emissions from these treated flowers was conducted using GC–MS (Supplementary Fig. S13).

**Figure 4:**
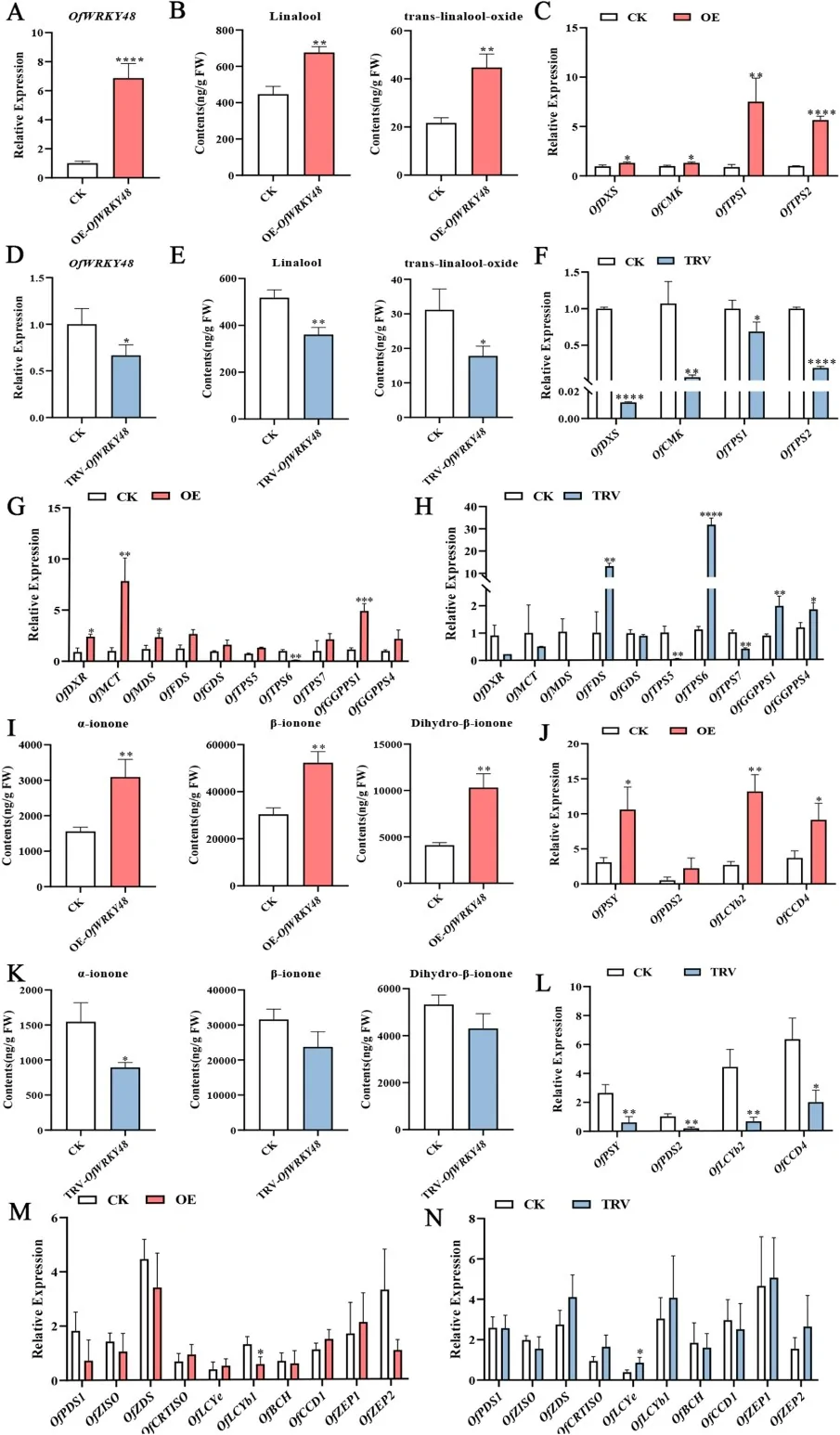
Functional verification of *OfWRKY48* by transient transgenic experiments in *O. fragrans* flowers. (A) Gene expression level of *OfWRKY48* in overexpressed strain; (B) Linalool and its oxides contents in overexpressed strain of *OfWRKY48*; (C) Expression levels of selected MEP pathway genes in overexpressed strain of *OfWRKY48*; (D) Gene expression level of *OfWRKY48* in silenced strain; (E) Linalool and its oxides contents in silenced strain of *OfWRKY48*; (F) Expression levels of selected MEP pathway genes in silenced strain. (G) Expression levels of the nonselected MEP pathway genes in overexpressed strain of *OfWRKY48*; (H) Expression levels of the nonselected MEP pathway genes in silenced strain of *OfWRKY48*; (I) α-ionone, β-ionone, and dihydro-β-ionone contents in overexpressed strain of *OfWRKY48*; (J) Expression levels of selected carotenoid biosynthesis pathway genes in overexpressed strain of *OfWRKY48*; (K) α-ionone, β-ionone, and dihydro-β-ionone contents in silenced strain of *OfWRKY48*; (L) Expression levels of the selected carotenoid biosynthesis pathway genes in silenced strain of *OfWRKY48*; (M) Expression levels of nonselected carotenoid biosynthesis pathway genes in overexpressed strain of *OfWRKY48*; (N) Expression levels of the nonselected carotenoid biosynthesis pathway genes in silenced strain of *OfWRKY48.* Data were presented as mean ± SD from three independent experiments, each performed with three biological replicates. Statistical significance relative to the control was determined by Student’s *t*-test: ^*^P < 0.05; ^**^P < 0.01; ^***^P < 0.001; ^****^P < 0.0001.

The contents of linalool and its oxides were significantly elevated in the OfWRKY48-overexpressing flowers compared with those in the control. Consistent with this, transcript levels of MEP pathway genes, including *OfDXS* (1-deoxy-D- xylulose-5-phosphate synthase), *OfDXR* (1-deoxy-D-xylulose-5- phosphatereductoisomerase), *OfMCT* (2-C-methyl-D-erythritol 4-phosphate cytidylyltransferase), *OfMDS* (2-C methyl-D-erythritol 2, 4-cyclodiphosphate synthase), *OfFDS* (farnesyl diphosphate synthase), *OfGGPPS1* (Geranylgeranyl diphosphate synthase 1), *OfCMK*, *OfTPS1* and *OfTPS2*, were markedly upregulated in OfWRKY48-overexpressing flowers, Conversely, VIGS-mediated suppression of OfWRKY48 led to reduced terpenoid accumulation and downregulation of key MEP pathway genes, such as *OfDXS*, *OfCMK*, *OfTPS1*, *OfTPS2*, as well as *OfTPS5* and *OfTPS7* (Fig. 4A–H). Notably, *OfDXS*, *OfCMK*, *OfTPS1* and *OfTPS2* exhibited pronounced transcriptional changes in response to OfWRKY48 modulation, suggesting their potential regulation by this transcription factor.

We further assessed the influence of OfWRKY48 on the synthesis of the volatile compounds α-ionone, β-ionone, and dihydro-β-ionone, as well as on the expression of key carotenoid metabolic genes. Overexpression of OfWRKY48 significantly promoted the production of these ionones and concurrently upregulated the transcript levels of *OfPSY* (phytoene synthase), *OfPDS2* (phytoene desaturase 2), *OfLCYb2* (lycopene beta-cyclase 2), and *OfCCD4* (carotenoid cleavage dioxygenase 4). In contrast, silencing of OfWRKY48 repressed the production of these compounds and downregulated the transcript levels of *OfPSY, OfPDS2*, *OfLCYb2*, and *OfCCD4* (Fig. 4I–N). Among these, *OfPSY*, *OfPDS2*, and *OfLCYb2* are likely responsible for supplying abundant precursor carotenoids, such as α-carotene and β-carotene, while *OfCCD4* efficiently catalyzes the cleavage of these carotenoids into fragrant aromatic compounds.

To evaluate the overall effect of this dual regulation on carotenoid accumulation, we quantified total carotenoid contents and documented floral phenotypes. We found that while OfWRKY48 overexpression promoted aroma compound production, it simultaneously led to a significant reduction in total carotenoid levels and visibly lighter petal coloration. Conversely, OfWRKY48 silencing increased carotenoid accumulation and resulted in darker floral pigmentation (Supplementary Fig. S14). These results demonstrated that OfWRKY48 coordinately upregulated both carotenoid biosynthesis and cleavage genes, thereby shifting metabolic flux toward volatile aroma production and establishing a trade-off between scent emission and pigment deposition.

### OfWRKY48 directly binds and activates the promotors of *OfTPS1*, *OfCMK*, and *OfLCYb2*

A subset of metabolic pathway genes, including *OfDXS*, *OfCMK*, *OfTPS1*, *OfTPS2*, *OfPSY*, *OfPDS2*, *OfLCYb2*, and *OfCCD4* displayed coordinated expression patterns with *OfWRKY48*, suggesting a potential regulatory role by this transcription factor. To investigate this hypothesis, promoter regions of these genes were subjected to computational motif analysis via the PlantCARE (https://bioinformatics.psb.ugent.be/webtools/plantcare/html/). The analysis revealed that W-box motifs—canonical binding sites for WRKY transcription factors—were exclusively present in the promoters of *OfDXS*, *OfCMK*, *OfTPS1*, *OfTPS2*, *OfPDS2*, *OfLCYb2*, and *OfCCD4*. Notably, no W-box motifs were detected in the promoters of *OfPSY* (Supplementary Fig. S15), suggesting divergent regulatory mechanisms for these genes despite their coexpression with OfWRKY48.

**Figure 5:**
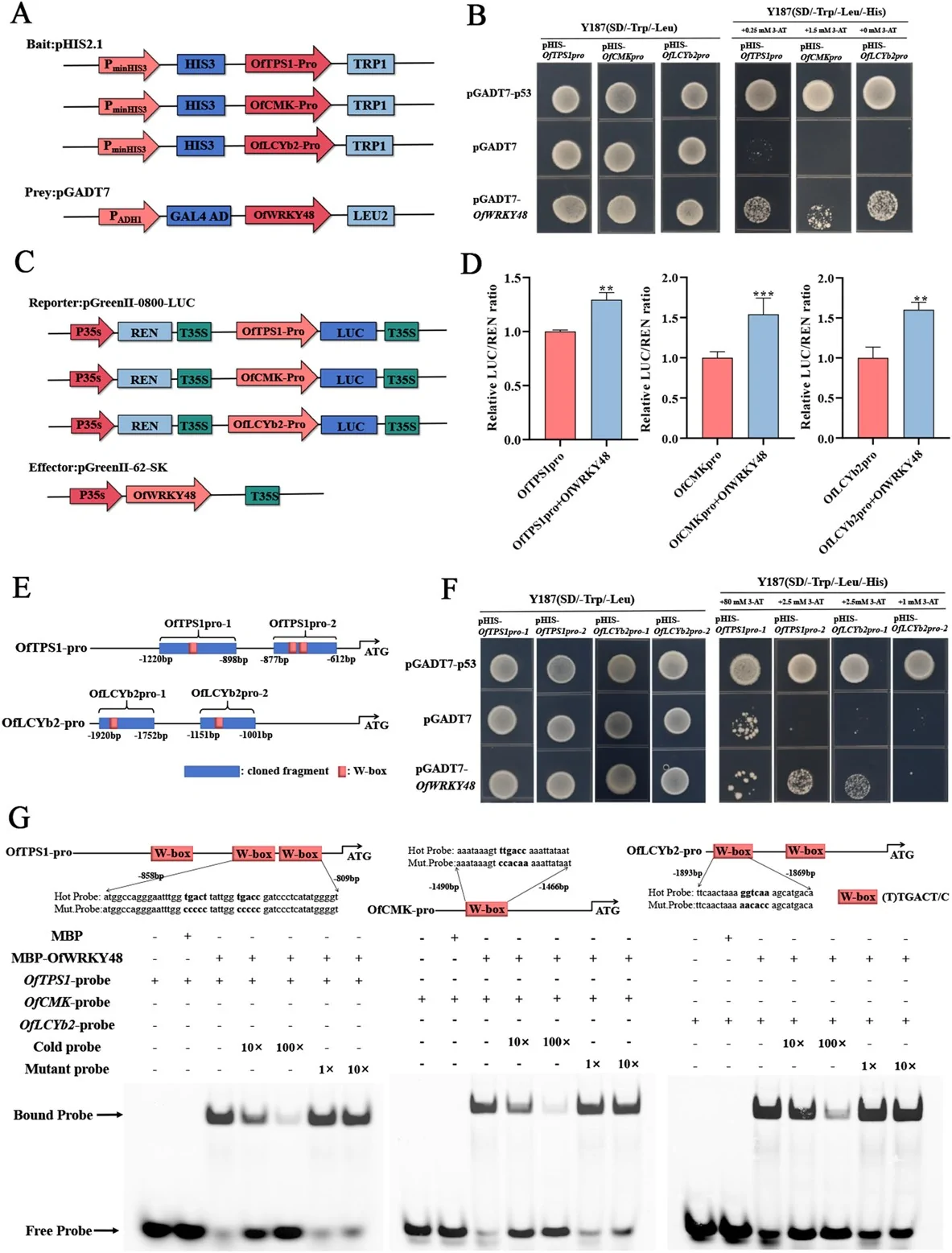
Interaction of the OfWRKY48 protein with the promoters of *OfTPS1, OfCKM* and *OfLCYb2.* (A) Schematic diagram of vector construction for the Y1H assay. (B) Y1H display the direct interaction between OfWRKY48 and *OfTPS1pro, OfCMKpro*, and *OfLCYb2pro*. (C) Schematic diagram of vector construction for the dual-luciferase assay. (D) Dual-luciferase assay shows relative OfWRKY48 activation of *OfTPS1pro, OfCMKpro*, and *OfLCYb2pro*. (E) Schematic of the promoter fragments used in Y1H assays. (F) Y1H assays indicate that OfWRKY48 directly binds to the promoter fragments *OfTPS1pro-2* and *OfLCYb2pro-1*. No interaction was detected with the *OfTPS1pro-1* or *OfLCYb2pro-2* fragments. (G) OfWRKY48 binds directly to the *OfTPS1pro, OfCMKpro*, and *OfLCYb2pro*. In EMSA experiments, Mut indicates probes with site-directed mutations. Competitor reactions used a 100× molar excess of unlabeled probe. Arrows mark shifted complexes and free probes. Data were presented as mean ± SD from three independent experiments, each performed with three biological replicates. Statistical significance relative to the control was determined by Student’s *t*-test: ^*^P < 0.05; ^**^P < 0.01; ^***^P < 0.001; ^****^P < 0.0001.

Y1H analysis demonstrated that cotransformation of OfWRKY48-AD with the promoters of *OfCMK*, *OfTPS1*, or *OfLCYb2* cloned into pHIS2.1 enabled robust growth of Y187 yeast cells on SD/-Trp/-Leu/-His medium supplemented with 3-AT. In contrast, no growth was observed in negative controls, including cotransformation of pGADT7 empty vector with the promoter constructs, or between OfWRKY48-AD and the promoters of *OfDXS*, *OfTPS2*, *OfPDS2*, or *OfCCD4* (Fig. 5A and B, Supplementary Fig. S16). Consistent with these findings, LUC reporter assays revealed that OfWRKY48 significantly enhanced the promoter activity of *OfTPS1*, *OfCMK*, and *OfLCYb2*, which was evident both from markedly elevated LUC/REN ratios and from direct visualization of the luminescence signals (Fig. 5C and D; Supplementary Fig. S17). These LUC assays further confirmed the lack of direct transactivation by OfWRKY48 on the *OfCCD4* promoter (Supplementary Fig. S18), suggesting an indirect regulatory mechanism despite the observed upregulation in functional studies.

To delineate the precise *cis*-elements underlying this transactivation, we performed a promoter segment analysis. Given that the *OfTPS1* and *OfLCYb2* promoters harbor multiple W-boxes, each was subdivided into discrete fragments (pro-1 and pro-2) for Y1H interaction assays. OfWRKY48 specifically interacted with the *OfTPS1*pro-2 and *OfLCYb2pro*-1 fragment but not with *OfTPS*1pro-1 or *OfLCYb2*pro-2, thereby pinpointing the functional W-boxes to the *OfTPS1*pro-2 and *OfLCYb2*pro-1 regions (Fig. 5E and F).

EMSA validation conclusively demonstrated direct interactions between OfWRKY48 and target DNA sequences. The OfWRKY48 coding sequence was cloned into the prokaryotic expression vector pMal-C2X to generate MBP-OfWRKY48, which was expressed in *Escherichia coli* Rosetta2 (DE3). The MBP fusion protein was affinity-purified with Dextrin Beads 6FF and used for interaction assays with *OfTPS1*, *OfCMK*, and *OfLCYb2* (Supplementary Fig. S19). Specifically, OfWRKY48 bound with high affinity to a 50-bp biotin-labeled probe derived from the *OfTPS1* promoter, which contains two tandem W-box elements at positions −841 to −837 bp and −830 to −826 bp. To distinguish the binding specificity of these two adjacent W-boxes, single-point mutation analysis was performed. Mutation of the first W-box abolished the binding signal, whereas mutation of the second had no effect, confirming that OfWRKY48 specifically binds to the first W-box (Supplementary Fig. S20). OfWRKY48 also bound with high affinity to a 25-bp probe from the *OfCMK* promoter (harboring a single W-box at −1481 to −1476 bp) and a 25-bp probe from the *OfLCYb2* promoter (harboring a single W-box at −1883 to −1878 bp), as evidenced by distinct shifted bands in EMSA autoradiograms (Fig. 5F). Binding specificity was further confirmed through competition assays: unlabeled wild-type probes effectively abolished complex formation at 10- to 100-fold molar excess, whereas mutated probes lacking intact W-box sequences failed to compete. OfWRKY48 has the potential to regulate all three targets (*OfCMK, OfTPS1, OfLCYb2*) in both cultivar groups, as the binding sites are present. The primary difference is its expression level: it is highly expressed in high-scent ‘Albus’ cultivars, leading to strong activation of both pathways, and lowly expressed in ‘Aurantiacus’ cultivars, resulting in low activation.

To ensure the general applicability of our mechanistic findings, we analyzed the sequences of *OfTPS1*, *OfCMK*, and *OfLCYb2* across all five cultivars. No fixed nucleotide differences were observed between the ‘Albus’ and ‘Aurantiacus’ groups in the coding sequences of these three genes, nor in the promoter regions of *OfTPS1* and *OfLCYb2*. A group-linked 10-bp InDel was identified in the *OfCMK* promoter; however, it was located far from any known *cis*-regulatory motifs, including the W-box element characterized in this study (Supplementary Fig. S21). Therefore, the protein functions and core promoter architectures relevant to OfWRKY48 binding were conserved, supporting the relevance of our regulatory model across cultivars.

**Figure 6:**
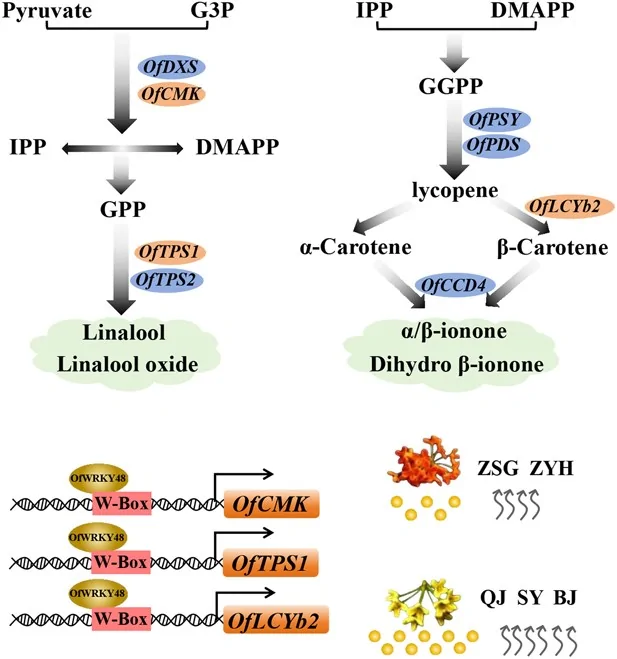
Schematic model of OfWRKY48-regulated scent biosynthesis in *O. fragrans*. Due to its higher expression level, the TF OfWRKY48 enhances volatile terpenoid production in the high-scent cultivar ‘Albus’ compared to the low-scent ‘Aurantiacus’ by directly activating the transcription of key genes (*OfCMK, OfTPS1, OfLCYb2*; orange ellipses) via promoter W-box elements, and indirectly modulating other pathway genes (*OfDXS, OfTPS2, OfPSY, OfPDS, OfCCD4*; blue ellipses). This regulation drives two major biosynthetic branches: (i) the monoterpene pathway from pyruvate/G3P to IPP/DMAPP (via *OfDXS/OfCMK*) and then to GPP, which is converted by *OfTPS1/OfTPS2* into linalool, with linalool subsequently oxidized to linalool oxides by cytochrome P450 enzymes; and (ii) the carotenoid-derived pathway from IPP/DMAPP to GGPP, lycopene (via *OfPSY*/*OfPDS*), α−/β-carotene (via *OfLCYe*/*OfLCYb*), and finally to α-ionone, β-ionone, and dihydro-β-ionone via *OfCCD4* cleavage. Higher OfWRKY48 expression thus amplifies the entire pathway, resulting in greater volatile emission (wavy arrows) in ‘Albus’. Bold arrows denote simplified biosynthesis; thin straight arrows indicate transcriptional activation. QJ (Qingjian), SY (Suiyin), BJ (Baijian), ZSG (Zhushagui), ZYH (Zhuangyuanhong).

We demonstrated that OfWRKY48 functioned as a master transcriptional regulator, directly activating terpenoid biosynthesis by binding to the promoters of core pathway genes. It not only directly activated three key targets—*OfCMK*, *OfTPS1*, and *OfLCYb2*—but also modulated the expression of other critical genes, including *OfDXS, OfTPS2, OfPSY, OfPDS,* and *OfCCD4*. The divergent scent profiles between ‘Albus’ and ‘Aurantiacus’ cultivars were primarily due to differing OfWRKY48 expression levels: high expression in ‘Albus’ led to robust pathway activation and high volatile terpenoid production, while low expression in ‘Aurantiacus’ resulted in abundant nonvolatile carotenoid accumulation (Fig. 6; Supplementary Fig. S14).

## Discussion

OfWRKY48 distinguishes itself from previously characterized floral scent regulators by operating as a metabolic coordinator rather than a pathway-specific activator. In *O. fragrans*, OfWRKY3 and OfWRKY33 primarily target individual carotenoid cleavage or linalool synthase genes [11, 12], whereas OfWRKY48 simultaneously engages three regulatory nodes: the MEP pathway (*OfCMK*), monoterpene synthesis (*OfTPS1*), and carotenoid flux (*OfLCYb2*). This multitiered architecture enables OfWRKY48 to synchronize precursor supply with terminal volatile formation—a control logic that mirrors principles of metabolic flux management observed in microbial systems but rarely documented in plant-specialized metabolism. Such coordinated regulation may represent an evolutionary solution to avoid metabolic bottlenecks that would arise from activating only downstream steps without commensurate precursor support.

### An integrated DEG and WGCNA approach identifies OfWRKY48 as a key transcriptional regulator

The identification of OfWRKY48 exemplifies a tiered filtering strategy that addresses a central challenge in nonmodel plant systems: distinguishing genuine regulatory hubs from coexpression noise. While WGCNA alone can identify modules correlated with metabolic traits, its integration with stringent DEG analysis (|log₂FC| > 2, FDR < 0.01) and tissue-specific expression profiling effectively reduced an initial pool of 213 transcription factors to a single high-confidence candidate with validated functional relevance. This approach is particularly critical in floral scent research, where transcriptional variation among cultivars often reflects complex combinations of developmental, environmental, and genetic factors rather than simple on–off switches. The success of this strategy in pinpointing OfWRKY48 underscores its potential for discovering master regulators in other nonmodel aromatic crops.

While integrated multi-omics approaches have successfully identified regulatory genes in other species—such as elucidating flavor loss in tomato [49] or aroma compound metabolism in kiwifruit [39]—applying such strategies in nonmodel plants requires robust frameworks to move beyond simple gene correlations and uncover genuine regulatory hubs. Building upon existing genomic resources for *O. fragrans* [5, 13, 50–52], we therefore designed a multitiered filtering strategy to systematically pinpoint key transcriptional regulators.

Our approach integrated DEGs analysis with WGCNA. We first identified 121 transcription factors differentially expressed in the high-scent ‘Albus’ group (Supplementary Table S7). Independently, WGCNA pinpointed 128 transcription factors within modules that were strongly correlated with the biosynthesis of linalool and ionones (Supplementary Table S9). The convergence of these two lists provided a high-confidence pool of candidates linked to the scent phenotype.

Subsequently, we applied a series of biological relevance filters to narrow down the pool to 58 candidate transcription factors. From these, eight highly expressed candidates were selected for further validation. RT-qPCR analysis across different cultivars and developmental stages further refined this list to six final candidates, including OfWRKY48. Its expression pattern not only corroborated the transcriptome data but also showed marked enrichment in ‘Albus’ flowers compared to ‘Aurantiacus’ group and other plant tissues (Fig. 2E, Supplementary Fig. S10).

The successful identification of OfWRKY48 underscores the power of combining DEG and WGCNA in nonmodel species to filter out background noise and highlight central regulatory nodes within coexpression networks. The generated datasets and the remaining candidate transcription factors provide a valuable resource for future studies aimed at elucidating the regulatory mechanisms of other aroma compounds in *O. fragrans*.

### OfWRKY48 coordinately regulates the biosynthesis of diverse floral volatiles in *O. fragrans*

WRKY transcription factors constitute a novel plant-specific protein family that plays important roles in growth and metabolism, including seed germination and maturation, seed and bud dormancy, leaf senescence, flowering time, phytohormone signaling, and stress and defense responses [53–57]. Accumulating evidence points to the involvement of WRKY transcription factors in the regulatory networks of secondary metabolism [58–60]. However, their specific roles in orchestrating multiple, distinct terpenoid pathways within a single biological context—such as floral scent production—remain underexplored.

The regulatory architecture of OfWRKY48 reveals an unexpected level of functional integration within the WRKY family. Unlike WRKY transcription factors that typically act on single biosynthetic branches—such as VvWRKY70 repressing norisoprenoid biosynthesis in grape [36] or GhWRKYs regulating flavonoid metabolism in cotton [58]—OfWRKY48 exerts dual direct control over both monoterpene and apocarotenoid pathways through distinct promoter binding events. Notably, its direct targets (*OfCMK*, *OfTPS1*, *OfLCYb2*) span three functionally distinct layers: upstream precursor biosynthesis, terminal volatile synthesis, and carotenoid branch-point flux [31]. The indirect regulation of *OfDXS*, *OfPSY*, *OfPDS2*, and *OfCCD4* further suggests a hierarchical regulatory cascade, potentially involving intermediate transcription factors or chromatin-level changes. This multilayered control strategy likely enables fine-tuned adjustments to metabolic output that single-target regulation cannot achieve—a design principle with significant implications for metabolic engineering.

Notably, OfWRKY48 did not directly bind promoters of several other affected genes (e.g. *OfDXS, OfPSY, OfPDS2, OfCCD4*), suggesting the involvement of indirect regulatory mechanisms. Such modular regulation is also seen in *lilac*, where SoNAC72 activates SoMYB44 to control anthocyanin biosynthesis [61]. We propose two nonmutually exclusive models: firstly, hierarchical regulation, whereby OfWRKY48 activates intermediate transcription factors that in turn regulate downstream structural genes; secondly, chromatin remodeling, where OfWRKY48 acts as a pioneer factor to make promoter regions of indirect targets more accessible to other transcriptional activators.

Our findings also provide a molecular explanation for the observed coenhancement of linalool and apocarotenoids in specific ‘Albus’ cultivars. Rather than operating in isolation, OfWRKY48 appears to synchronize precursor flux and terminal volatile synthesis across pathways, highlighting its potential as a strategic target for metabolic engineering or fragrance-oriented breeding in *O. fragrans*.

### OfWRKY48 coordinates the synthesis of floral scent and defense volatiles

Our results position OfWRKY48 as a key regulator that coordinates the synthesis of both terpenoid and apocarotenoid volatile compounds. This dual function integrates the production of floral scent for pollinator attraction with the emission of defense volatiles, suggesting a central role in balancing reproductive and defensive functions. Similarly, the bHLH transcription factor LaMYC7 enhances linalool production and disease resistance in *lavender* [62]. The synchronized regulation of linalool and β-ionone, in particular, points to a sophisticated mechanism for optimizing volatile blends for specific ecological outcomes.

This coordinated regulation is exemplified by a ‘push–pull’ dynamic that fine-tunes plant–insect interactions. Linalool acts as a primary attractant for key pollinators such as bees, moths, and hoverflies [8, 63, 64], while also serving direct and indirect defensive roles by deterring herbivores like aphids and spider mites and recruiting their natural enemies. Similarly, β-ionone contributes to defense by reducing herbivore feeding and attracting predators [65]. The ecological relevance of this co-regulation is underscored by its precise spatiotemporal control: the peak emission of these volatiles in *O. fragrans* flowers between 9 a.m. and 12 p.m. aligns perfectly with the foraging activity of primary pollinators like *Vespa nigrithorax* and *Apis florea* [66, 67]. This ensures that pollinator attraction is efficient while maintaining a constant defensive backdrop.

The regulatory influence of OfWRKY48 extends to a critical metabolic trade-off through its promotion of *OfCCD4*, a hub carotenoid cleavage dioxygenase. By directing carotenoid substrates into the production of apocarotenoid volatiles like β-ionone, this mechanism may concurrently reduce floral pigmentation (shifting from orange to pale yellow/white), thereby prioritizing olfactory signaling over visual cues. Such a trade-off may represent an adaptive strategy in contexts where scent is a more reliable pollinator guide. This function mirrors findings in other species, such as poplar, where PyWRKY48 overexpression alters pigment dynamics to enhance stress tolerance [68], indicating that WRKY transcription factors commonly act as metabolic switches to reallocate resources. A hierarchical regulatory cascade, ATAF1-AP2/ACE1-PAP1/MYB21L2, was also uncovered to fine-tune floral color (anthocyanin) and scent (linalool) dynamics during flower development in *F. hybrida* [69].

The capacity of OfWRKY48 to manage this complex portfolio of traits stems from its ability to modulate a broad transcriptional network. Our transient overexpression and VIGS experiments revealed a ‘ripple effect’ on noncanonical targets, suggesting its regulatory influence extends beyond direct promoter binding. Potential mechanisms include the indirect activation of downstream transcription factors or chromatin remodeling. To fully dissect this hierarchical control, future work should employ Y2H, Co-IP, and GST pull-down assays to systematically identify OfWRKY48-interacting partners, such as chromatin modifiers or secondary transcription factors. Elucidating these interactions will be crucial for understanding how OfWRKY48 coordinates the activation of spatially and temporally linked genes to achieve a balanced volatile output.

## Conclusion

This study identifies OfWRKY48 as a key transcriptional regulator that coordinates the production of both terpenoid and apocarotenoid volatiles, the major components of *O. fragrans* floral scent. Our findings reveal that OfWRKY48 directly activates promoters of core genes in the MEP pathway (*OfCMK*) and a key terpene synthase (*OfTPS1*), while simultaneously directly upregulating a critical carotenoid pathway gene (*OfLCYb2*). This coordinated action synchronizes precursor supply with branching points in distinct metabolic pathways, ultimately enhancing the synthesis of linalool and its oxides, as well as ionone derivatives. The regulatory model uncovered here—where a single transcription factor orchestrates a complex metabolic network through both direct and indirect mechanisms—provides a new, more integrated understanding of how floral scent is regulated. As such, OfWRKY48 represents a promising target for the molecular breeding of aroma and pigment traits in horticultural crops.

### Plant materials

Flowers from five distinct *O. fragrans* cultivars were utilized. The light-yellow-to-white ‘Albus’ group included ‘Suiyin’ (SY), ‘Qingjian’ (QJ), and ‘Baijian’ (BJ). The orange ‘Aurantiacus’ group comprised ‘Zhushagui’ (ZSG) and ‘Zhuangyuanhong’ (ZYH). All floral samples were harvested at full bloom between 10 a.m. and 11 a.m. from the grounds of Huazhong Agricultural University, Hubei, China.

Following volatile collection, samples were immediately snap-frozen in liquid nitrogen and stored at −80°C for subsequent analysis. *N. benthamiana* plants were cultivated in a greenhouse under controlled conditions: 16 h light / 8 h dark photoperiod, 24°C.

### Volatile and free aroma compounds analysis by GC–MS

To profile floral volatiles, HS-SPME coupled with GC–MS was performed as previously outlined [5]. Briefly, petal tissues (0.2 g FW) were homogenized with the internal standard (methyl nonanoate) and then underwent HS-SPME. The extraction was carried out for 30 min at 55°C using a DVB/Carboxen/PDMS fiber.

For nonvolatile (free) aroma compounds, a solvent extraction protocol with methyl tert-butyl ether (MTBE) was followed [7]. Frozen flower tissue (2 g) was ground in liquid nitrogen and extracted twice with 10 ml of a pentane/diethyl ether mixture (1:1, v/v) at 16°C for 30 min each. Pooled extracts were held at −20°C overnight. The supernatant was then collected, concentrated under a gentle nitrogen stream to ~2 ml, spiked with cyclohexanone (internal standard), and dehydrated over anhydrous MgSO₄ prior to GC analysis.

Both HS-SPME and solvent extracts were separated on a DB-5MS capillary column with helium carrier gas (1 ml/min constant flow). The oven temperature was programmed appropriately. The mass spectrometer was operated using electron ionization (70 eV) and scanned masses from ^*^m/z^*^ 40–450. Identification of compounds followed two criteria: first, matching the acquired spectra against the NIST 2017 database (requiring >80% similarity); second, comparison of their experimental retention indices with literature Kovats values. Quantification was achieved through the use of internal standards.

### Principal component analysis

To validate the overall structure and reproducibility of the volatile metabolome data, a PCA was performed. The analysis was based on the OAVs of the major volatile compounds (OAV >100) identified across the five cultivars. The data matrix was autoscaled (mean-centered and unit-variance scaled) prior to PCA. PCA was executed in R (v4.2.0) using the prcomp function, with results visualized as score and loading plots via the ggplot2 package. Variance explained by each principal component is indicated in the corresponding figure.

### Transcriptome analysis and weighted gene coexpression network analysis

Transcriptome analysis was conducted on fully bloomed flowers from each cultivar. Total RNA was extracted using the RNeasy Plant Mini Kit (Qiagen). Three biological replicates per sample were sequenced on an Illumina HiSeq 2500 platform. After adapter trimming and quality filtering, clean reads were aligned to the reference genome [48] and assembled using TOPHAT (v2.1.1) and CUFFLINKS (v2.2.1). Gene expression was quantified and normalized as Fragments Per Kilobase of transcript per Million mapped reads (FPKM). Raw sequencing data are available in the NCBI SRA under BioProject accession PRJNA1300594.

DEGs were identified using DESeq2 in R. Genes with |log₂ fold change| > 2 and an adjusted *P*-value (FDR) <0.01 (Benjamini–Hochberg procedure) were deemed significant. Low-expression genes (FPKM <10 across all samples) were filtered out prior to analysis.

For WGCNA, the WGCNA R package was used. After removing low-expression genes (FPKM <10), a subset of highly variable genes (coefficient of variation, CV > 0.5) was selected for network construction. An unsigned topological overlap matrix (TOM) was built using a soft-power threshold (β) of 10. Modules were detected with a minimum size of 30 genes, and a merge cut height of 0.25. Module eigengenes (first principal components) were correlated with volatile and free terpenoid contents to identify biologically relevant modules.

### Gene isolation, phylogenetic tree construction, and multiple sequence alignment

The coding sequence (CDS) of OfWRKY48 was amplified via reverse transcription PCR from RNA extracted from *O. fragrans* flowers. The amplified product was cloned into a pGEM-T vector (Promega) and sequenced bidirectionally to confirm integrity. For phylogenetic analysis, homologous WRKY protein sequences were retrieved from NCBI. A maximum-likelihood tree was then constructed in MEGA 7.0 based on the aligned sequences, with branch support evaluated from 1000 bootstrap replicates.

### RNA extraction and quantitative real-time PCR

Total RNA was isolated from various tissues (roots, stems, leaves) and floral development stages (bud, initial bloom, full bloom, late bloom) of *O. fragrans* ‘Suiyin’ using a Trizol-based kit. The extracted RNA was reverse-transcribed into cDNA, and subsequent real-time PCR analysis was performed on a Roche LightCycler 480 platform. Gene expression was quantified via the 2^(-ΔΔCt) method [5].

### Subcellular localization

The OfWRKY48 CDS (stop codon removed) was fused into the pSAT6-EYFP-N1 vector under the control of the 35S promoter. The construct was transformed into Agrobacterium GV3101. Bacterial cultures (OD_600_ = 0.6–0.8) were resuspended in infiltration buffer (10 mM MES, 10 mM MgCl₂, 20 μM acetosyringone, pH 5.6), incubated for 2 h at 28°C in the dark, and infiltrated into leaves of 28-day-old *N. benthamiana* plants. Fluorescence was observed by confocal microscopy 60 h after infiltration.

### Transcriptional activation assay

The CDS of *OfWRKY48* were inserted into the pGBKT7 vector to generate the recombinant pGBKT7-OfWRKY48 plasmid. The recombinant plasmid, positive control (pGBKT7-p53 + pGADT7-T), and negative control (pGBKT7) vectors were transformed into the yeast (*S. cerevisiae*) strain AH109 Gold, the yeast strains were cultured on SD (−Trp) medium and SD (−Trp/-His/−Ade) medium to determine the transactivation activity.

### Transient transformation of *O. fragrans* flowers

Transient overexpression and silencing in *O. fragrans* flowers were performed. The OfWRKY48 coding sequence was inserted into PK7WG2D for overexpression, while a fragment of its 3’ UTR was ligated into the pTRV2 vector for VIGS. Both constructs were transferred into *A. tumefaciens* strain GV3101. After preparation, bacterial suspensions were vacuum-infiltrated into fully open flowers (0.06 MPa, 10 min). Floral volatiles were profiled by HS-SPME–GC–MS at 60 h postinfiltration [5].

### Y1H assay

Promoters of candidate genes were cloned into pHIS2.1, while the OfWRKY48 CDS was cloned into pGADT7. Plasmids were cotransformed into yeast strain Y187. Protein–DNA interactions were assessed by culturing the transformed yeast cells on dropout media (SD/-Trp/-Leu/-His) supplemented with varying concentrations of 3-amino-1,2,4-triazole (3-AT).

### Dual-luciferase reporter assay

The OfWRKY48 CDS in pGreenII62-SK served as the effector. Promoters of *OfTPS1*, *OfCMK*, and *OfLCYb2* were cloned into pGreenII0800-LUC as reporters. Constructs were coinfiltrated into *N. benthamiana* leaves. Luciferase activities were measured 3 days postinfiltration using a dual-luciferase assay kit (Promega), with six biological replicates. Primers are in Supplementary Table S13.

### Electrophoretic mobility shift assay

The OfWRKY48 CDS was inserted into pMal-C2X to produce an MBP-OfWRKY48 fusion protein in *E. coli* Rosetta2 (DE3). Protein expression was induced with 0.5 mM IPTG at 16°C for 14 h and purified using Dextrin Beads 6FF. Biotin-labeled probes containing W-box motifs from target promoters were synthesized. To distinguish the binding specificity of the two adjacent W-box motifs in the *OfTPS1* promoter, probes with single-point mutations in each W-box core sequence were also synthesized (Supplementary Fig. S20). Binding reactions and competition assays with unlabeled wild-type or mutated probes were performed as described [5].

### Statistical analysis

Results are expressed as mean ± SD from a minimum of three independent experiments, each comprising three biological replicates (*n* = 3). Statistical comparisons between two groups were performed using an unpaired Student’s *t*-test, with significance levels denoted as follows: ^*^P < 0.05; ^**^P < 0.01; ^***^P < 0.001；^****^P < 0.0001. To compare measurements of multiple experiment designs with each other, univariate analysis of variance (ANOVA) followed by *post hoc* Tukey’s test was performed.

#### Accession numbers

The gene sequences generated in this study have been deposited in the NCBI database (https://www.ncbi.nlm.nih.gov/). The accession number for *OfWRKY48* is PX310283.

## Supplementary Material

Web_Material_uhag167

## Data Availability

Raw sequencing reads of all *O. fragrans* accessions reported in this study have been deposited into the public database of the National Center of Biotechnology Information (NCBI) BioProject under the accession number PRJNA1300594. All data in this study are provided in the article and its supplementary materials.

## Notes

This statement, following the corresponding author line above: The author responsible for distribution of materials integral to the findings presented in this article in accordance with the policy described in the Instructions for Authors (https://mts-hortres.nature.com/cgi-bin/main.plex) is R.Z.
